# Proposition of a Silica Nanoparticle-Enhanced Hybrid Spin-Microcantilever Sensor Using Nonlinear Optics for Detection of DNA in Liquid

**DOI:** 10.3390/s151024848

**Published:** 2015-09-25

**Authors:** Wen-Hao Wu, Ka-Di Zhu

**Affiliations:** Key Laboratory of Artificial Structures and Quantum Control (Ministry of Education), Department of Physics and Astronomy, Shanghai Jiao Tong University, Shanghai 200240, China; E-Mail: hao0792@sina.cn

**Keywords:** hybrid spin sensor, nonlinear optics, detection of DNA

## Abstract

We theoretically propose a method based on the combination of a nonlinear optical mass sensor using a hybrid spin-microcantilever and the nanoparticle-enhanced technique, to detect and monitor DNA mutations. The technique theoretically allows the mass of external particles (ssDNA) landing on the surface of a hybrid spin-microcantilever to be detected directly and accurately at 300 K with a mass responsivity 0.137 Hz/ag *in situ* in liquid. Moreover, combined with the nanoparticle-enhanced technique, even only one base pair mutation in the target DNA sequence can be identified in real time accurately, and the DNA hybridization reactions can be monitored quantitatively. Furthermore, *in situ* detection in liquid and measurement of the proposed nonlinear optical spin resonance spectra will minimize the experimental errors.

## 1. Introduction

Highly sensitive and selective DNA detection has attracted extensive attention for its importance in clinical diagnostics, treatment and various genome projects. For several decades, there has been a flurry of activities involving micro-/nano-mechanical systems as precision micro-/nano-mechanical sensors to detect DNA, proteins and other biomolecules [1,2,3,4,5,6,7,8,9,10,11,12,13,14,15,16,17]. All of these detection methodologies have outstanding performance. However, there still exist some defects: (i) strict environment for detection: most of these accurate sensors [9,11,15] operate at extremely low temperature and high vacuum, such as T= 4 K/40 K and $10^{-5}$ torr, while the natural environment where biological processes occur is aqueous solutions and ambient temperature; (ii) *ex situ* measurements in air and vacuum: in order to circumvent the low quality (Q) problem in liquid, the dip and rinse measure approach [3,8,16,18] was used. However, the main limitation of the dip and rinse approach is that unspecific binding and contamination occur during desiccation; and (iii) mass resolution: though the mass detection limits have been recently pushed down to the yoctogram range, *i.e.*, the mass of a single proton can be measured [17], practically, the mass change between the single base pair mismatched DNA and the normal DNA (about 1 Dalton) is still too small to determine directly and accurately.

Besides, nitrogen vacancy (NV) centers in diamond are amongst the most promising implementations of quantum bits for quantum information processing [19] and nanoscale field sensors [20]. Recently, high quality (Q) factor single-crystal diamond (SCD) mechanical resonators [21,22] have been demonstrated as hybrid spin-micro-/nano-mechanical systems. Teissier *et al.* [23] presented results on the resolved sideband regime for NV spin-microcantilever coupling at room temperature. Due to the NV’s long quantum coherence time and diamond’s low mechanical losses, the implementation of this hybrid spin-nanomechanical system, especially in mass sensors based on an SCD cantilever, would represent a significant progress in nanotechnology.

For the sake of quickly discerning whether DNA mutations occur, we theoretically propose the application of a silica nanoparticle-enhanced hybrid spin-microcantilever as a sensor and applied the optical pump-probe technique for detection in the present article. Particularly, the optical pump-probe technique has been demonstrated experimentally [24], compared to the electrical output technique [25], to avoid the heating effect during the measurement. Besides, the microcantilever’s resonance frequency can be detected through the well-established optical NV spin readout technology [26]. Though a lot of optical mass sensors based on micro-/nano-mechanical systems have been proposed so far [11,12], all of these schemes remain in the linear optics regime. Using the nonlinear optical spectrum in the present article can reduce the effect of detection noise and offer better performance over the linear optical spectrum [27]. Moreover, due to the NV’s long quantum coherence time and diamond’s low mechanical losses at room temperature, the proposed spin-microcantilever sensor can perform well at 300 K and will allow detection in real time in liquid. These properties eliminate the drawback of *ex situ* measurements in air and vacuum. Overall, via measuring the microcantilever’s resonance frequency shift before and after the DNA hybridization reaction, we can easily obtain the mass of external particles landing on the surface from the nonlinear optical spectrum. Furthermore, quantitative information on the DNA hybridization mechanism might be obtained. Meanwhile, whether the target DNA mutated is also determined.

## 2. Model and Theory

**Figure 1:**
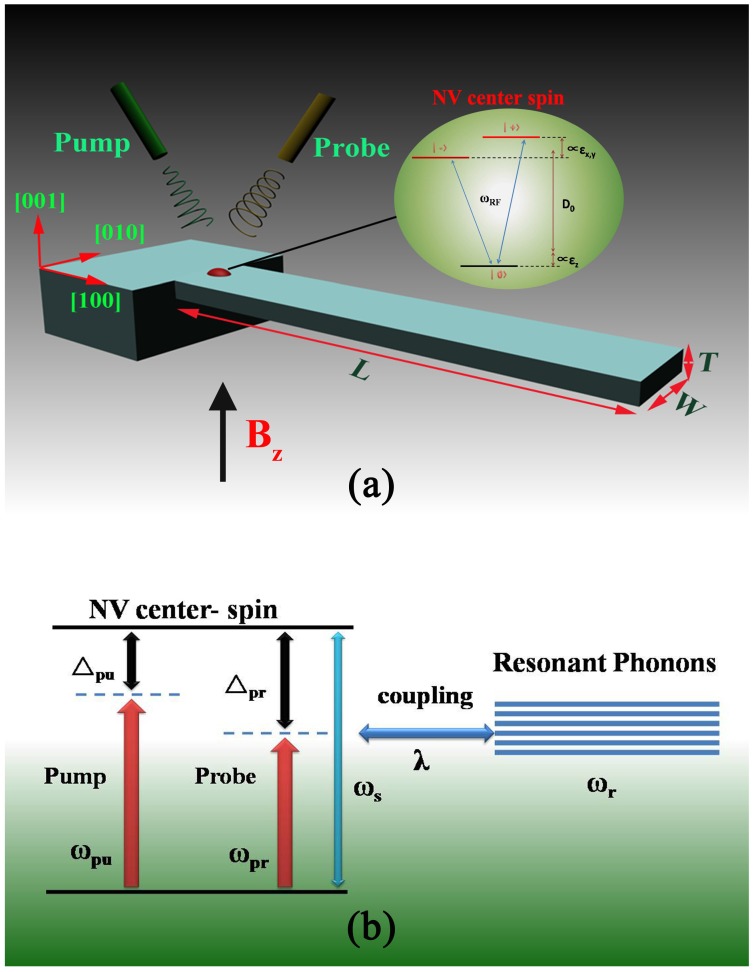
(**a**) Schematic diagram of the hybrid spin-microcantilever system in the presence of a strong pump field and a weak probe field. The inset is the energy level diagram of a nitrogen vacancy (NV) center spin. ${\left[\varepsilon_{i}\right]}_{i=x,y,z}$ are the diagonal components of the strain tensor defined in the NV’s basis (note that we have neglected shear); (**b**) The energy level diagram of the coupling between the NV center spin and the microcantilever.

We consider a system consisting of a high-Q SCD cantilever with a single NV spin center embedded at the base, as the coupling is maximized when the NV spin is at the base [23,28], in the simultaneous presence of a strong pump microwave field $\omega_{pu}$, a weak probe microwave field $\omega_{pr}$ and an external magnetic field along the *z* axis. The physical prototype is illustrated in Figure 1a. The motion of the cantilever along the *z* axis is quantized and described by the Hamiltonian $H_{r}=\hbar \omega_{r}a^{+}a$, with $\omega_{r}$ as the frequency of the fundamental bending mode and *a* and $a^{+}$ as the corresponding annihilation and creation operators.

For the NV center spin in diamond, the schematic diagram of energy levels is shown in the inset of Figure 1a. Here, we model strain by an effective electric field in which strain-induced displacements of the atoms alter the electron density in the crystal, resulting in local electric fields [29,30]. Therefore, the energies can be expressed as a function of the normalized beam displacement *X* when the NV is in the presence of a DC magnetic field $B_{z}$ aligned closely to its symmetry axis (ℏ=1) [28]:

(1) $$E_{0}≃0;E_{\pm }\left(X\right)≃D_{0}+g_{∥}X\pm \sqrt{{\left(\gamma_{NV}B_{z}\right)}^{2}+{\left(g_{⊥}X\right)}^{2}}$$

where $D_{0}$ is the crystal-field splitting ($D_{0}\approx 2.87$ GHz), $\gamma_{NV}$ is the gyromagnetic ratio ($\gamma_{NV}\approx 2.8$ MHz·G${}^{-1}$) and $g_{∥}$ and $g_{⊥}$ are the axial and transverse strain couplings to the zero point motion of the resonator. Additionally, we applied a magnetic field $B_{z}=26$ G along the NV axis to suppress the effects of transverse strain [23], as shown in Equation (1), such that our discussion can be restricted to the two-level subspace spanned by ∣ 0> and ∣ +>, and mixing of ∣ -> and ∣ +> by transverse strain can be neglected [23,28]. Therefore, the Hamiltonian of the NV center spin can be described as $H_{s}=\hbar \omega_{s}S^{z}$, where $\omega_{s}=2\pi \left(D_{0}+\gamma_{NV}B_{z}\right)$, and the spin operator can be characterized by the spin operators $S_{\pm }$ and $S^{z}$.

The schematic diagram of energy levels of the coupling between the NV spin and the cantilever’s vibration is shown in Figure 1b. Therefore, the spin-cantilever interaction can be described by [28,31,32] $H_{s-r}=\hbar \lambda \left(a+a^{+}\right)S^{z}$, where *λ* is the coupling of spin to phonon ($\lambda =2\pi g_{∥}$). As a strong pump microwave field $\omega_{pu}$ and a weak probe microwave field $\omega_{pr}$ are simultaneously applied to this coupled spin-cantilever system, the NV center spin via spin-flip interacts with them. We treat these microwave fields classically. The Hamiltonian of the NV center spin transitions through microwaves is described as [33] $H_{s-p}=-\mu_{B}\left(S^{+}B_{pu}e^{-i\omega_{pu}t}+S^{-}B_{pu}^{*}e^{i\omega_{pu}t}\right)-\mu_{B}\left(S^{+}B_{pr}e^{-i\omega_{pr}t}+S^{-}B_{pr}^{*}e^{i\omega_{pr}t}\right)$, where $\mu_{B}$ is the Bohr magneton, $\omega_{pu}\left(\omega_{pr}\right)$ is the frequency of the pump field (probe field) and $B_{pu}\left(B_{pr}\right)$ is the slowly varying envelope of the pump field (probe field). Therefore, we obtain the total Hamiltonian of the coupled spin-micromechanical cantilever in the presence of two microwave fields:

(2) $$\begin{array}{c} H=H_{r}+H_{s}+H_{s-r}+H_{s-p}=\hbar \omega_{r}S^{z}+\hbar \omega_{s}aa^{+}+\hbar \lambda \left(a+a^{+}\right)S^{z}\\ -\mu_{B}\left(S^{+}B{}_{pu}e^{-i\omega_{pu}t}+S^{-}B_{pu}^{*}e^{i\omega_{pu}t}\right)-\mu_{B}\left(S^{+}B{}_{pr}e^{-i\omega_{pr}t}+S^{-}B_{pr}^{*}e^{i\omega_{pr}t}\right). \end{array}$$

In a rotating frame at the pump field frequency $\omega_{pu}$, the total Hamiltonian of the system reads as follows:

(3) $$\begin{array}{c} H=\hbar \Delta_{pu}S^{z}+\hbar \omega_{r}aa^{+}+\hbar \lambda \left(a+a^{+}\right)S^{z}-\hbar \left(\Omega S^{+}+\Omega^{*}S^{-}\right)\\ -\mu_{B}\left(S^{+}B{}_{pr}e^{-i\delta t}+S^{-}B_{pr}^{*}e^{i\delta t}\right), \end{array}$$

where $\Delta_{pu}=\omega_{s}-\omega_{pu}$ is the detuning between the spin and the pump microwave field, $\Omega =\mu_{B}B_{pr}/\hbar$ is the pump microwave Rabi frequency and $\delta =\omega_{pr}-\omega_{pu}$ is the detuning between the probe microwave field and the pump microwave field.

Applying the Heisenberg equation of motion for operators $S^{z}$, $S^{-}$ and *N* and introducing the damping and noise terms phenomenologically, we obtain the corresponding quantum Langevin equations as follows [34,35]:

(4) $$\frac{dS^{z}}{dt}=-\Gamma_{1}\left(S^{z}+1/2\right)+i\Omega \left(S^{+}-S^{-}\right)+\left(\frac{i\mu_{B}B_{pr}}{\hbar }\right)\left(S^{+}e^{-i\delta t}-S^{-}e^{i\delta t}\right)\text{,}$$

(5) $$\frac{dS^{-}}{dt}=-\left[i\left(\Delta_{pu}+\lambda N\right)+\Gamma_{2}\right]S^{-}-2i\Omega S^{z}-2i\mu_{B}B_{pr}e^{-i\delta t}S^{z}/\hbar +{\hat{F}}_{n}\text{,}$$

(6) $$\frac{d^{2}N}{dt^{2}}+\gamma_{r}\frac{dN}{dt}+\omega_{r}^{2}N=-2\omega_{r}\lambda S^{z}+\hat{\xi }\text{.}$$

where $N=a^{+}+a$ is the position operator, $\Gamma_{1}$ and $\Gamma_{2}$ are the electronic spin relaxation rate and dephasing rate and $\gamma_{r}=\omega_{r}/Q$ is the intrinsic decay rate of high-Q SCD cantilever. ${\hat{F}}_{n}$ is the *δ*-correlated Langevin noise operator, which has zero mean $\langle {\hat{F}}_{n}\rangle =0$ and obeys the following correlation functions $\langle {\hat{F}}_{n}\left(t\right){\hat{F}}_{n}^{+}\left(t^{\prime }\right)\rangle ∼\delta \left(t-t^{\prime }\right)$.

The motion of the nanomechanical resonator is affected by the thermal bath of Brownian and non-Markovian stochastic processes [35]. The quantum effects on the resonator are only observed in the limit of a very high quality factor, which obeys $Q=\omega_{r}/\gamma_{r}\gg 1$. The Brownian noise operator can be modeled as Markovian with the decay rate $\gamma_{r}$ of the resonator mode. Therefore, the Brownian stochastic force has zero mean value $\langle {\hat{\xi }}_{n}\rangle =0$ that can be characterized as: $\langle {\hat{\xi }}^{+}\left(t\right)\hat{\xi }\left(t^{\prime }\right)\rangle =\frac{\gamma_{r}}{\omega_{r}}\int \frac{d\omega }{2\pi }\omega e^{-i\omega (t-t^{\prime })}\left[1+\coth\left(\frac{\hbar \omega }{2\kappa_{B}T}\right)\right]$ [36], where $k_{B}$ and *T* are the Boltzmann constant and the temperature of the reservoir of the coupled system, respectively.

To go beyond weak coupling, the Heisenberg operator can be rewritten as the sum of its steady-state mean value and a small fluctuation with zero mean value: $S^{z}=S_{0}^{z}+\delta S^{z},S^{-}=S_{0}^{-}+\delta S^{-}$ and $N=N_{0}+\delta N$. Following standard methods from quantum optics, we derive the steady-state solutions to Equations (4)–(6) by setting all of the time derivatives to zero. Since the driving fields are weak, but classical coherent fields, we will identify all operators with their expectation values and drop the quantum and thermal noise terms [37]. Simultaneously, inserting these operators into the Langevin equations, Equations (4)–(6), and neglecting the nonlinear term, we can obtain two equation sets about the steady-state mean value and a small fluctuation. The steady-state equation set consisting of $N_{0}$ and $S_{0}^{-}$ is related to the population inversion ($w_{0}=2S_{0}^{z}$) of the exciton, which is determined by:

(7) $$\left(w_{0}+1\right)\left[{\left(\Delta_{pu0}-\lambda_{0}^{2}w_{0}/\omega_{r0}\right)}^{2}+1\right]+2\Omega_{R}^{2}w_{0}=0$$

For the equation set of small fluctuation, we make the ansatz [33] $\langle \delta S^{z}\rangle =S_{+}^{z}e^{-i\delta t}+S_{-}^{z}e^{i\delta t}$, $\langle \delta S^{-}\rangle =S_{+}e^{-i\delta t}+S_{-}e^{i\delta t}$ and $\langle \delta N\rangle =N_{+}e^{-i\delta t}+N_{-}e^{i\delta t}$. Solving the equation set and working to the lowest order in $B_{pr}$, but to all orders in $B_{pu}$, we can obtain $S_{+}$, which corresponds to the linear optical susceptibility and $S_{\_}$ corresponding to the nonlinear optical susceptibility, as follows:

(8) $$S_{+}=\frac{Gw_{0}+DS_{0}^{*}\left(2\Omega_{R}+\lambda_{0}\xi S_{0}\right)}{\Omega_{R}D\left(2\Omega_{R}+\lambda_{0}\xi S_{0}\right)-GC},$$

(9) $$S_{-}=\frac{3}{G^{*}D^{*}}\left(\frac{2\omega_{r0}w_{0}\xi^{*}\lambda_{0}^{2}}{C+\delta_{0}}+2\right)\left(\frac{w_{0}}{C^{*}}-\frac{w_{0}}{C+\delta_{0}}\right).$$

Actually, we detect NV spin transitions through a well-established [26] optical NV spin readout to perform optical electron spin resonance (ESR). The nonlinear spin resonance spectrum is exactly the average population of the spin states measured in the majority of experiments [23,28], which can be given by:

(10) $$S_{-}^{z}=\frac{-1}{-\delta_{0}+i\Gamma_{1}}\left(\Omega S_{+}^{*}-\Omega^{*}S_{-}-\frac{\mu_{B}B_{pr}}{\hbar }S_{0}^{-}\right),$$

where

$C=\Delta_{pu0}-i-\delta_{0}-\lambda_{0}^{2}w_{0}/\omega_{r0}$, $D=\Delta_{pu0}+i+\delta_{0}-\lambda_{0}^{2}w_{0}/\omega_{r0}$,
$\;\;\;S_{0}=\Omega_{R}^{2}w_{0}\xi \lambda_{0}^{2}/\omega_{r0}\left(C+\delta_{0}\right)$, $S_{0}^{*}=\Omega_{R}^{2}w_{0}\xi \lambda_{0}^{2}/\omega_{r0}\left(D-\delta_{0}\right)$,
$\;\;\;G=\left(\delta_{0}+i\Gamma_{1}/\Gamma_{2}\right)-\frac{2}{D}\left(S_{0}^{*}+\Omega_{R}^{2}\right)+\frac{2}{C}\left(S_{0}+\Omega_{R}^{2}\right)$,

and the auxiliary function $\xi (\omega_{pr})$ is given by:

(11) $$\xi \left(\omega_{pr}\right)=\frac{\omega_{r0}^{2}}{\omega_{r0}^{2}-i\delta_{0}\gamma_{ro}-\delta_{0}^{2}},$$

where $\delta_{0}=\delta /\Gamma_{2},\Omega_{R}=\Omega /\Gamma_{2},\lambda_{0}=\lambda /\Gamma_{2},\omega_{r0}=\omega_{r}/\Gamma_{2},\gamma_{r0}=\gamma /\Gamma_{2},\Delta_{pu0}=\Delta_{pu}/\Gamma_{2}$and $\Gamma_{1}=2\Gamma_{2}$. As the nonlinear optical Kerr coefficient is proportional to the real part $Re(\chi^{\left(3\right)})$ of the nonlinear optical susceptibility [33], the probe Kerr spectrum can be obtained conveniently.

The hybrid cantilever can be described by harmonic oscillators with an effective mass $m_{eff}$, a spring constant *k* and a resonance frequency $\omega_{r}=\sqrt{k/m_{eff}}$. Atoms or molecules landing on the surface of the microcantilever can significantly increase the total mass of the microcantilever resonator, which will simultaneously reduce the resonance frequency. Mass sensing is based on monitoring the frequency shift $\Delta \omega_{r}$ of $\omega_{r}$. Expanding Δm in Taylor series, the relationship between $\Delta \omega_{r}$ with the increased mass Δm can be expressed as:

(12) $$\Delta m=\frac{2m_{eff}}{\omega_{r}}\left(\frac{1}{{\left(1-\Delta \omega_{r}/\omega_{r}\right)}^{2}}-1\right)\approx \Delta m^{\prime }=\frac{2m_{eff}}{\omega_{r}}\Delta \omega_{r}=R^{-1}\Delta \omega_{r}$$

where $\Delta m^{\prime }$ is the increased mass acquired with the linear approximation, and $R={\left(\frac{2m_{eff}}{\omega_{r}}\right)}^{-1}$ is defined as the mass responsivity. The relative error caused by linear approximation is defined as $|\Delta m-\Delta m^{\prime }|/\Delta m$.

## 3. Results and Discussion

In the following, we consider an experimentally-realistic hybrid spin-microcantilever at an ambient temperature of 300 K to display our nonlinear optical mass sensor: the dimensions of the cantilever are 10 *μ*m long, 3.5 *μ*m wide and 0.2 *μ*m thick. It has a fundamental frequency $\omega_{r}/2\pi =6.659$ MHz and a quality factor $Q=10^{6}$ [21,22], and the effective mass of the microcantilever $m_{eff}=2.42\times 10^{-11}g$ [23]. With feasible experimental parameters, the decay rate of the microcantilever is $\gamma_{r}=\omega_{r}/Q$, and the longitudinal spin relaxation of the NV center $T_{1}=10$ ms [38,39].

**Figure 2:**
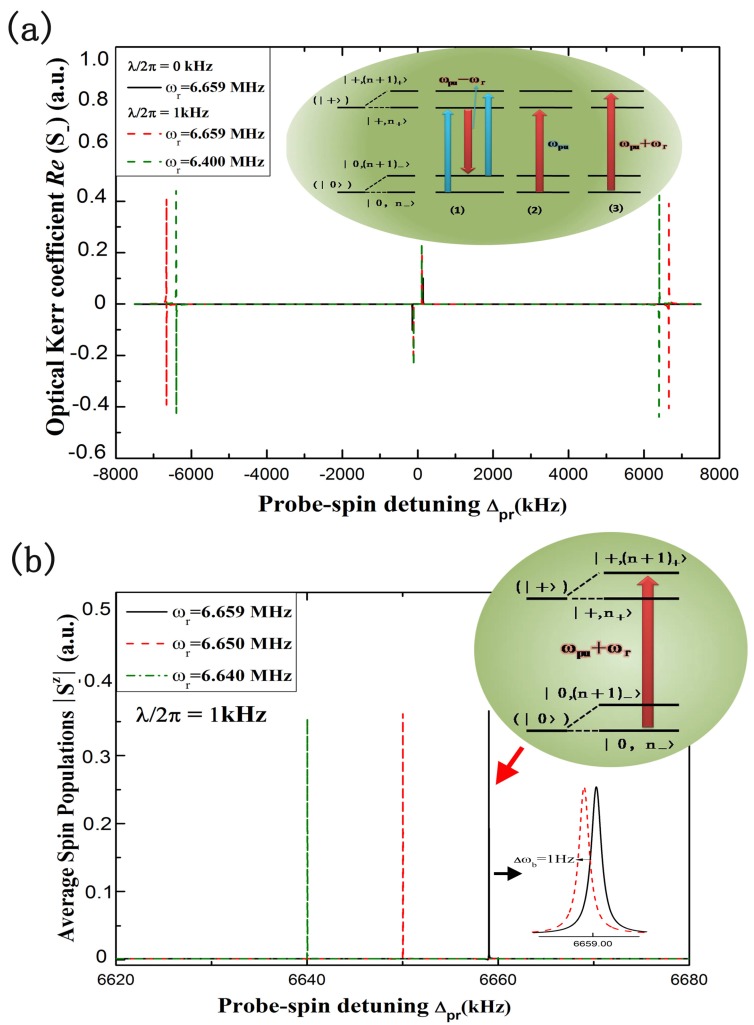
(**a**) The optical Kerr coefficient as a function of the detuning of the probe field from the spin resonance with two vibrational frequencies of the hybrid spin-microcantilever $\omega_{r}/2\pi =6.659$ MHz and $\omega_{r}/2\pi =6.400$ MHz for $\Omega_{pu}^{2}=100$ (kHz), $\Delta_{pu}=0$ and λ/2π=1 kHz. Additionally, the new features in the spectrum shown are identified by the corresponding transition between the dressed states of NV center spin, as shown in the inset; (**b**) The nonlinear spin resonance spectrums $\langle S_{-}^{z}\rangle$ as a function of $\Delta_{pr}$ corresponding to different vibrational frequencies (all of the other parameters are the same as (**a**). The upper inset shows the underlying physical mechanism for these characteristic peaks. The bottom inset is a partially enlarged view of the characteristic peak.

Naturally, we should illustrate how to weigh the mass of accreted particles landing onto the surface of the hybrid spin-microcantilever using the above optical pump-probe technique. Mass sensing is based on detecting the frequency shift of the hybrid spin-microcantilever. The external particles depositing on the surface of hybrid spin-microcantilever will lead to a small frequency shift. Then, the particles can be weighed by determination of a small frequency shift via using Equation (14). The main question is how to determine the original frequency (without depositing particles on the surface) of hybrid spin-microcantilever. Here, we provide a scheme to measure the original frequency of the hybrid spin-microcantilever via the probe nonlinear Kerr coefficient spectrum. Figure 2a shows the optical Kerr coefficient $Re(\chi^{\left(3\right)})$ as a function of the probe detuning $\Delta_{pr}$ when the pump field is resonant with the spin ($\Delta_{pu}=0$). In the absence of coupling between the NV center spin and microcantilever (λ/2π=0 kHz, $\omega_{r}=6.659$ MHz), corresponding to the black solid curve, there are no new features in the spectrum. With the coupling turned on (λ/2π=1 kHz, $\omega_{r}=6.659$ MHz), it is obvious, from the red dashed curve in Figure 2a, that two new sharp peaks appear at $\Delta_{pr}=\pm 6.659$ MHz, corresponding to the resonator frequency of the hybrid spin-microcantilever. To demonstrate its validity, we set the hybrid spin-microcantilever’s resonator frequency to be $\omega_{r}=6.400$ MHz. Then, the two sharp peaks appear at the point $\Delta_{pr}=\pm 6.400$ MHz, corresponding to the resonator frequency, as shown by the green-dashed curve. Such a phenomenon can be interpreted by a dressed state picture in which the original energy levels of the NV center spin have been dressed by the phonon mode of the microcantilever, as shown in the inset [11,12]. The physical origin of this result is due to mechanically-induced coherent population oscillation, which makes quantum interference between the resonator and the beat of the two optical fields via the localized exciton when the probe-pump detuning is equal to the resonator frequency. Surely, when fixing the pump field detuning $\Delta_{pu}=0$ and scanning the probe frequency across the NV spin frequency $\omega_{s}$ in the spectrum, then we can easily and exactly obtain the vibration frequency of the hybrid spin-microcantilever from the nonlinear Kerr spectrum.

In addition, the nonlinear optical results can always be detected by the well-established optical NV spin readout technology [26]. For better experimental guidance, the nonlinear spin resonance spectrum $\langle S_{-}^{z}\rangle$ as a function of the detuning is also presented. As shown in Figure 2b, when all other parameters are the same as in Figure 2a, characteristic peaks also appear in the nonlinear spin resonance spectrum corresponding to the vibrational frequency $\omega_{r}$. The inset of Figure 2a shows the underlying physical mechanism for these characteristic peaks. Therefore, this nonlinear spin resonance spectrum can also reveal the vibrational frequency of the microcantilever as the nonlinear Kerr spectrum does.

After the original frequency of the hybrid spin-microcantilever has been determined via the nonlinear spin resonance spectrum, we can demonstrate our optical mass sensing based on the hybrid spin-microcantilever and proceed to detect the target DNA. We assume that the added mass is distributed uniformly on the hybrid microcantilever, and the added mass does not affect the spring constant *k* of the resonator (as $m_{add}\ll m_{eff}$), as does the coupling strength, but instead, the resonance frequency in the coupling term. In consequence, the mass responsivity of this hybrid microcantilever sensor is:

(13) $$R={\left(\frac{2m_{eff}}{\omega_{r}}\right)}^{-1}=0.137\;Hz/ag$$

where $m_{eff}=2.42\times 10^{-11}g$, $\omega_{r}=6.659$ MHz.

**Figure 3:**
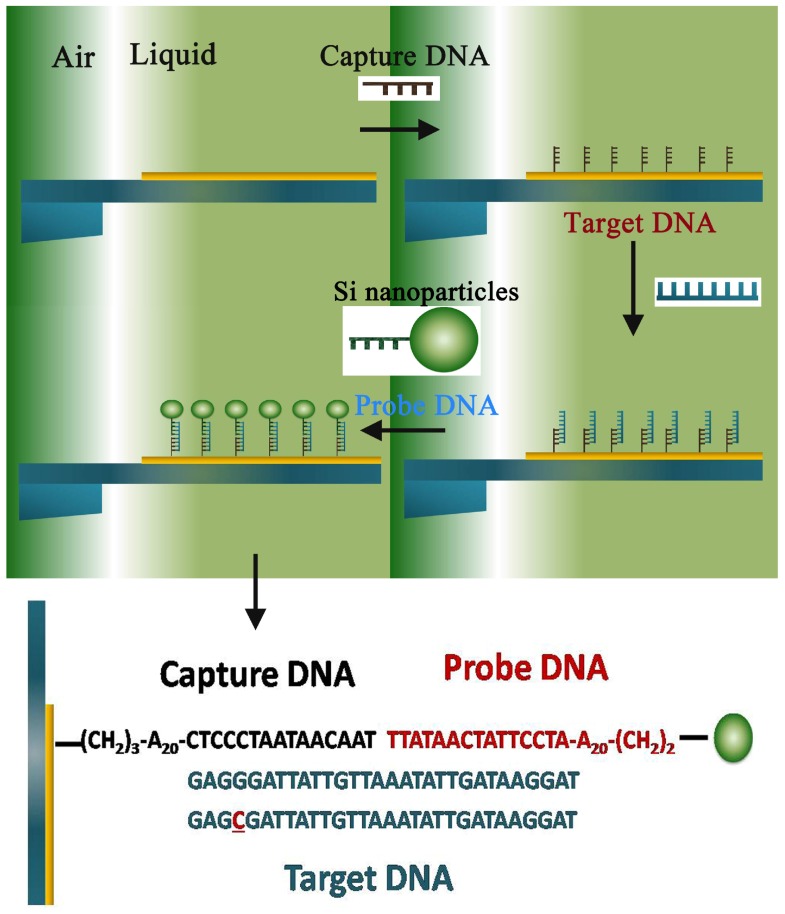
Proposed scheme of hybrid the spin-microcantilever based on *in situ* DNA detection using a nanoparticle probe in liquid and the sequences of capture DNA, target DNA, probe DNA and single base pair mismatched DNA.

In general, nanomechanical resonators in liquids exhibit a very low quality factor *Q*(1–10) as a consequence of the viscous damping. However, when only the top surface of the microcantilever is wetted in the liquid (as shown in the inset of Figure 3), the vibration plane is parallel to the liquid-air interface and the beam is resonating in air; the quality factor of the resonator is not degraded by viscous damping and the drag of the fluid on the largest section [13,14,40]. To this, the hybrid spin-microcantilever still can perform detection in liquid with excellent mass responsivity R=0.137 Hz/ag. Meanwhile, the noise processes in our theoretical simulations should be mentioned. Actually, a microcantilever in fluid fluctuates with respect to the rest position mainly due to the random impacts of the surrounding molecules. In the same way, the cantilever dissipates the stored mechanical energy through its interaction with the surrounding molecules that constitute in this case the thermal bath. Thermomechanical noise determines the ultimate detection limits of the micromechanical sensor [41,42]. For a microcantilever oscillating at one of its natural frequencies $\omega_{r}$, the mean-square frequency due to thermal noise is given by [43]:

(14) $$\langle \Delta \omega_{th,r}^{2}\rangle \approx \frac{\Delta \omega_{b}k_{B}T}{4A^{2}\pi^{2}m_{eff}Q\omega_{r}}$$

where $k_{B}$ is Boltzmann’s constant, *T* is the environment temperature, $A=\sqrt{\hbar /(2m_{eff}\omega_{r})}$ is the oscillation amplitude and $\Delta \omega_{b}$ is the frequency bandwidth of the measurement characteristic peak, $\Delta \omega_{b}=1$ Hz, as shown in the bottom right corner inset in Figure 2b. The minimal mass can be detected by the microcantilever, given by [44]:

(15) $$\Delta m_{min}\approx \sqrt{\frac{m_{eff}\Delta \omega_{b}k_{B}T}{16A^{2}\pi^{2}Q\omega_{r}^{3}}}$$

When we set the quality factor $Q=10^{6}$ and T=300 K, the detectable minimum mass that has taken thermomechanical noise into account is about 1.02fg according to the above Equation. This performance is equal to that of a hybrid sensor with quality factor Q=240 working in air.

Therefore, the relatively modest value of detectable minimal mass measured at room temperature and under atmospheric pressure is $\Delta m_{min}=1.02\;fg$, which has taken the clamping losses and noise of experimental conditions into account. Furthermore, compared to the mass sensing based on the linear optical method, the use of a nonlinear optical spectrum may overcome the detection noise and offer better performance in the presence of detection noise [27].

For this assay, the capture DNA on the hybrid spin-microcantilever surface and the probe DNA conjugated with silica nanoparticles were designed specifically for the target DNA. Then, the detail simulative process schematic is shown at the top of Figure 3. Firstly, enzyme linked immunosorbent assay (ELISA) experiments were specifically designed to verify the microcantilever surface-DNA binding specificity and immobilized protein activity [13], so the microcantilever is functionalization. As the hybrid spin-microcantilever was partly immersed into the liquid and appropriate ambient conditions were met, the capture DNA would be immobilized on the surface. Next, the target DNA and the silica nanoparticle-enhanced probe DNA in solution with the optimal concentration were added in turn. Meanwhile, the vibrational frequency of the spin-microcantilever was measured after each step, and the hybridization reactions between every step were fully completed. To efficiently detect the target DNA using the silica nanoparticle-enhanced DNA assay, the size of silica nanoparticles is optimized by directly binding the silica nanoparticles through DNA hybridization [3,8]. Here, we set the diameter of the enhanced silica nanoparticle to be 150 nm.

**Figure 4:**
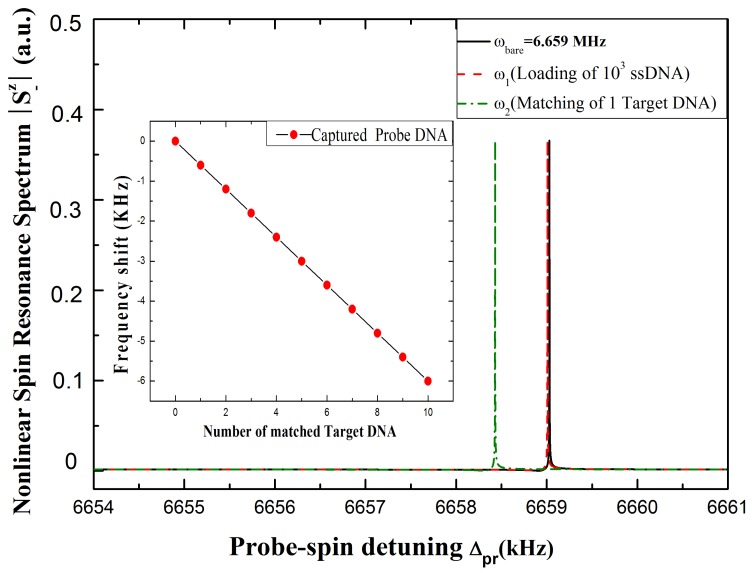
The nonlinear spin resonance spectrum with and without linking the ssDNA onto the surface of the hybrid spin-microcantilever. The black curve shows the initial resonance of the hybrid spin-microcantilever; the green-dashed curve indicates that after binding $10^{3}$ capture ssDNA; and the red-dashed curve indicates that after binding one normal target DNA and one silica nanoparticle-enhanced probe DNA are linked to the capture ssDNA. The left inset exhibits the relationship between the frequency shift of the hybrid spin-microcantilever and the numbers of linked silica nanoparticle-enhanced probe DNAs. The other parameters used are the same as Figure 2.

In order to analyze the assay, a reasonable assumption is made that there have been about $10^{3}$ capture DNA strands immobilized on the surface. The known mass of a single ssDNA molecule ($m_{DNA}=999$ kDaltons) and the mass loading on the spin-microcantilever could be $m_{1}=16.6ag$. According to Equation (16), the frequency shift is $\Delta \omega_{r1}=2.26$ Hz, corresponding to the green dashed curve shown in Figure 4. Then, as shown at the bottom of Figure 3, the sequence of normal target DNA strand is $5^{\prime }-GAGGGATTATTGTTAAATATTGATAAGGAT-$$3^{\prime }$. If the target DNA is normal, all of the hybridization reactions will complete and the silica nanoparticle-enhanced probe DNA will be linked on the other end of the target DNA through complementary interactions. Even if only one silica nanoparticle-enhanced probe DNA is linked, the mass added is about $m_{2}=4.13\;fg$ and the frequency shift is $\Delta \omega_{r2}=565$ Hz, corresponding to the red solid curve shown in Figure 4. On the contrary, if the target DNA has mutated even by just single base pair, $5^{\prime }-GAGCGATTATTGTTAAATATTGATAAGGAT-$ $3^{\prime }$, as shown at the bottom of Figure 3, the hybridization reactions will not carry on. Therefore, the silica nanoparticle-enhanced probe DNA will be absent, and the frequency shift will remain ($\Delta \omega_{r1}=2.26$ Hz). As a consequence, we propose to easily and precisely get the result whether or not the target DNA is mutated. Moreover, the left inset of Figure 3 shows the linear relationship between the resonance frequency shifts and the number of linked target DNA. The negative slope gives the mass sensitivity of the resonator. Combining this curve and the other condition, the concentrations of each ssDNA can be obtained in real time; thus, the DNA hybridization reactions can be monitored quantitatively.

For some of the DNA detection sensors, strict ambient environments (extremely low temperature and a vitally high vacuum) are needed. Fortunately, this hybrid spin-microcantilever sensor works well at T=300 K in liquid directly, the natural environment where biological processes occur. Simultaneously, the operating environment also avoids the drawback of *ex situ* measurements in air and vacuum of unspecific binding and contamination during desiccation. Besides, our optical pump-probe scheme can avoid the heating effect during the electrical measurement and will generate a beat wave to drive the mechanical resonator, which allows both the high and the low frequency of the mechanical resonator.

## 4. Conclusions

We have theoretically demonstrated that the hybrid spin-microcantilever system can be employed as a mass sensor in the nonlinear optical domain, operating at T=300 K in liquid directly. Because of the quantum interference between the vibration modes and the two optical fields, the mechanical resonance frequency can be determined conveniently and precisely from the nonlinear spin resonance spectrum. Besides, the heating and energy loss characterized by the electrical detections can be avoided, and the main limitation of *ex situ* measurements in air and vacuum, unspecific binding and contamination during desiccation, will be solved. Furthermore, this nonlinear optical mass sensor based on the hybrid spin-microcantilever can be applied to detect the mutated DNA quickly in real time and also can monitor the DNA hybridization reactions quantitatively due to its excellent characteristics in all optical domains. We expect that our nonlinear optical detection scheme can be implemented in current experiments.
